# Plasma exosomal lncRNA SLC16A1-AS1 as an early diagnostic biomarker for renal cell carcinoma

**DOI:** 10.3389/fonc.2026.1908708

**Published:** 2026-09-11

**Authors:** Yu Sun, Zhicheng Li, Chao Ren, Dan Wang, Xiaojun Zhu

**Affiliations:** Department of Urology, Affiliated Hospital of Inner Mongolia Medical University, Hohhot, Inner Mongolia, China

**Keywords:** diagnostic biomarkers, lncRNA SLC16A1-AS1, lncRNAs, plasma exosomes, renal cell carcinoma

## Abstract

**Purpose:**

Advanced renal cell carcinoma (RCC) has a poor prognosis due to metastasis, delayed diagnosis and drug resistance. Therefore, the early diagnosis of the disease is of vital importance, but currently there are no validated circulating blood biomarkers available for the prediction of early-stage renal cell carcinoma. This study aimed to identify plasma exosomal long non-coding RNAs (lncRNAs) as novel diagnostic biomarkers to support early RCC diagnosis.

**Methods:**

We obtained 5 mL plasma from every individual among 6 RCC patients and 6 healthy controls, alongside other 36 paired RCC tumor and adjacent non-tumor tissues. Plasma exosomes were isolated by ultracentrifugation and characterized via transmission electron microscopy (TEM), nanoparticle tracking analysis (NTA) and western blotting (WB). Differentially expressed exosomal lncRNAs were screened by next‐generation sequencing (NGS). Candidate lncRNAs were validated by qPCR in RCC cell lines and clinical tissues. Receiver operating characteristic (ROC) and the area under curve (AUC) analysis were used to evaluate the diagnostic efficacy. Kaplan–Meier analysis was applied to explore the prognostic value.

**Results:**

We successfully isolated and identified exosomes. By applying NGS, we successfully screened out five plasma exosomal lncRNAs—ENSG00000261253, FRRS1L, LINC02035, DNAJC7 and SLC16A1-AS1. Meanwhile, sequencing results further confirmed that these lncRNAs are significantly upregulated in plasma exosomes from RCC patients. QPCR was performed to detect the expression levels of five candidate lncRNAs in renal cell carcinoma cell lines as well as paired carcinoma and adjacent non-tumor tissues. Among these lncRNAs, only SLC16A1-AS1 was markedly upregulated in both cellular and tissue specimens. Based on 36 pairs of renal cell cancer tissue samples, the ROC curve was calculated, which revealed its AUC = 0.901, indicating excellent diagnostic efficacy and potential for early diagnosis of renal cell cancer. Kaplan–Meier analysis based on the TCGA database further demonstrated that both overall survival (OS) and disease-free survival (DFS) were significantly decreased in the high-expression group, suggesting its high expression predicts unfavorable patient prognosis.

**Conclusions:**

This study identifies plasma exosomal lncRNA SLC16A1-AS1 may serve as a preliminary potential non-invasive biomarker for RCC early diagnosis and prognostic monitoring. Future *in vivo*/vitro experiments will explore its functional mechanisms, providing theoretical basis for RCC treatment.

## Introduction

Renal cell carcinoma (RCC) is a predominant urological malignancy, contributing to 85% of renal cancers and 2–3% of adult malignant tumors (1). Located in the retroperitoneal cavity, RCC grows silently with atypical early clinical symptoms (2). Most early RCC patients remain asymptomatic, while only a small proportion develop hematuria, lumbago or abdominal masses (3). Conventional imaging modalities including abdominal ultrasound (US), computed tomography (CT) and Magnetic resonance imaging (MRI) are routinely used for renal tumor assessment, yet they have notable drawbacks for early-stage RCC diagnosis. Ultrasound often misses small intrarenal lesions and fails to distinguish tumor subtypes or benign-malignant status. Despite high diagnostic performance of CT, accurate differentiation between neoplastic lesions and RCC remains difficult. MRI also cannot reliably characterize renal tumors and frequently requires biopsy confirmation (4). RCC is insensitive to radiotherapy and chemotherapy, and surgical resection remains the primary therapeutic option (5). The 5-year survival rate is 72% for resectable early RCC, but declines to 12% for unresectable metastatic disease (6). Even with current diagnostic and therapeutic advances, 20–30% of RCC patients present distant metastasis at initial diagnosis (7). Therefore, effective early detection and diagnosis are essential to improve the survival of RCC patients.

Tumors proliferate and metastasize by remodeling the surrounding microenvironment, releasing internal components into the circulation. Accordingly, biofluids including blood, urine, tears and cerebrospinal fluid are ideal candidates for cancer screening (8). Exosomes are 30–150 nm lipid bilayer vesicles actively secreted by living cells and widely distributed in multiple body fluids such as plasma, urine, cerebrospinal fluid, saliva and tears (9), which carry proteins, lipids, and nucleic acids including lncRNAs, circular RNAs (CircRNAs) and microRNAs (MiRNAs) to regulate intercellular signal transduction, thereby modulating antigen presentation, cell proliferation and differentiation, tumor immunity, as well as tumor invasion and metastasis (10, 11). Exosomal mRNAs are consistent with those of primary tumors, implying their tumor cell origin and ability to reflect tumor features; thus, exosomes hold great potential as biomarkers for early cancer diagnosis and prognostic evaluation (12). LncRNAs are a class of RNA molecules that are longer than 200 nucleotides but do not participate in protein coding (13). Accumulating evidence demonstrates that lncRNAs function as oncogenes or tumor suppressors during tumor initiation and progression (14). Their biological roles depend on subcellular localization (15). Nuclear lncRNAs regulate histone modification, transcriptional activation, chromatin remodeling and alternative splicing (16). Cytoplasmic lncRNAs are involved in post-transcriptional and translational gene regulation via binding intracellular proteins and modulating mRNA metabolism through miRNAs (17). Collectively, aberrantly expressed lncRNAs regulate tumor initiation, progression and drug resistance through direct and indirect molecular interactions. LncRNAs thus serve as promising biomarkers for cancer diagnosis and prognostic evaluation, and represent novel therapeutic targets for tumor treatment.

Most reported plasma exosomal lncRNA biomarkers (e.g., lncARSR, AGAP2-AS1) are validated exclusively in advanced RCC to predict therapeutic resistance, with rare explorations for early asymptomatic patients (18). There are few literature reports on the study of lncRNAs in RCC plasma exosomes as early diagnostic markers. This study isolated plasma exosomes from patients with early-stage RCC and healthy controls. NGS was performed to screen differentially expressed lncRNAs in plasma exosomes between the two groups. Candidate lncRNAs were further validated at cellular and tissue levels. ROC curves were plotted and the corresponding AUC values calculated to assess their diagnostic efficacy. Additionally, Kaplan–Meier analysis was adopted to explore the correlation of candidate lncRNAs with patient survival outcomes, and this survival analysis was performed using the public TCGA RCC dataset (n=429 and n=430). We hypothesized that plasma exosomal lncRNA expression patterns differ markedly between early RCC patients and healthy controls, and that selected dysregulated exosomal lncRNA biomarkers could achieve satisfactory diagnostic performance for early RCC and correlate with patient clinical prognosis. Through the above research, we hypothesized that plasma exosomal lncRNAs differ between early RCC patients and healthy controls and that selected candidates may show diagnostic and prognostic relevance.

## Materials and methods

### Sample collection

RCC patients and healthy asymptomatic individuals were enrolled from the Department of Urology, Affiliated Hospital of Inner Mongolia Medical University between January 2024 and January 2026. A total of six plasma samples were collected from six RCC patients and six matched healthy controls forming the discovery cohort for next-generation sequencing; meanwhile, the 36 pairs of RCC tumor samples and adjacent non-tumor tissue samples used for subsequent validation were all derived from 36 independent patients who underwent surgical treatment for RCC, and did not include any subjects that overlapped with those in the discovery cohort. The clinical data of the participants, including age, gender, pathological type, TNM stage, and surgical method were recorded. All tissue samples were immediately frozen in liquid nitrogen and stored at -80 °C. The human renal cancer cell lines (ACHN, 786-O, 769-P) and the normal human renal tubular cell line (HK-2) were purchased from Servicebio. 786-O and 769-P are primary clear cell renal cell carcinoma (ccRCC) cell lines with VHL gene inactivation, isolated from primary human ccRCC lesions. ACHN was derived from malignant pleural effusion of metastatic RCC patient. HK-2 is an immortalized normal human proximal tubule epithelial cell line from healthy adult kidney tissue, serving as normal renal control cells. The study has been approved by the Ethics Committee of Inner Mongolia Medical University Affiliated Hospital (Approval Number: KY2024079), and the patients have signed the informed consent form. We acknowledge the limited statistical power caused by the small discovery cohort size, and we define our sequencing screening as a pilot study, with our findings validated in an independent tissue cohort to reinforce reliability.

### Inclusion and exclusion criteria

Inclusion and exclusion criteria for the renal cell cancer group: 1) Age between 20 and 80 years old; 2) Diagnosed by histopathological examination as the basis for confirmation, and confirmed as having renal cell cancer after surgery; 3) Surgical method was standard laparoscopic unilateral nephrectomy; 4) No previous anti-tumor treatment; 5) No concurrent cancer conditions; 6) Required to sign an informed consent form.

Inclusion and exclusion criteria for the healthy control group: 1) Age between 20 and 80 years old; 2) Undergone health examination before inclusion, ultrasound examination showed no kidney masses, no obvious discomfort or in a healthy state; 3) Required to sign an informed consent form. Exclusion criteria: 1) History of kidney diseases; 2) Concurrent other malignant tumors; 3) Long-term chronic disease history, long-term medication history, and clear family history of tumors.

### Exosome isolation and identification

Plasma samples (5mL) were collected from 6 renal cell cancer patients and 6 healthy controls. Fresh plasma samples are stored at 4 °C and processed within 1 hour. After pre-cooling, the plasma is centrifuged at 3000×g and 12000×g for 10 minutes each to remove cell debris and impurities, and the supernatant is stored at -80 °C. The frozen plasma is rapidly thawed in a water bath at 37 °C, then subjected to gradient centrifugation at 2000×g for 30 minutes and centrifugation at 10000×g at 4 °C for 45 minutes to remove large extracellular vesicles. The supernatant was filtered through a 0.45 μm sterile membrane and ultracentrifuged at 100000×g for 70 min at 4 °C. The exosome pellet was gently resuspended in pre-cooled 1×PBS for subsequent use. Isolated exosomes were identified by TEM, NTA, and WB. For TEM, exosomes were negatively stained with phosphotungstic acid and observed at 80.0 kV. Calibrated NTA detected exosome size distribution and concentration.

### Western blot

Exosomal proteins were extracted using Radio Immunoprecipitation Assay (RIPA) lysis buffer containing protease inhibitors. Exosome samples were mixed with pre-cooled RIPA buffer, and the supernatant was collected for the bicinchoninic acid (BCA) protein quantification assay. BCA Standard Curve and Sample Detection Concentration are presented in **Tables 1**, **2** respectively. A 5% stacking gel and a 10% separating gel were prepared, and approximately 20 μg of protein sample was loaded onto the gel in the designated order. Following electrophoresis, proteins were transferred to 0.22 μm PVDF membranes at a constant current of 400 mA for 80 min. The membranes were blocked with 5% skim milk powder at room temperature for 1.5 h and washed three times with TBST (10 min per wash). The primary antibodies against CD9 (Bioss, Cat. No. Bs-2489R), CD81 (Bioss, Cat. No. Bs-6934R), and TSG101 (Bioss, Cat. No. Bs-1365R) were diluted at a ratio of 1:2000 in 1% skim milk solution. Membranes were incubated with diluted primary antibodies overnight at 4 °C, followed by 1 h incubation at room temperature. After three TBST washes, membranes were incubated with goat anti-rabbit secondary antibody (Yike Biotechnology, Cat. No. YK2231, 1:10000 dilution) at room temperature for 1 h. After another three rounds of TBST rinsing, protein bands were visualized by chemiluminescence imaging using the Tanon 5200multi system, and the gray value of each band was quantified with GIS 1D software.

**Table 1:** BCA standard curve.

Standard sample absorbance	1.896	1.067	0.513	0.317	0.223	0.093	0
Standard sample concentration	2	1	0.5	0.25	0.125	0.0625	0

**Table 2:** Sample detection concentration.

Sample number	10	13	14	17	D	F	G	J
Measure the absorbance	1.999	2.704	1.268	2.268	2.850	1.642	1.818	2.443
Detect concentration	2.070	2.821	1.290	2.357	2.977	1.689	1.877	2.543

### Exosome RNA sequencing

Exosomal total RNA was isolated using the QIAGEN miRNeasy Plasma Kit. RNA quality and concentration were determined by Nanodrop OneC, LabChip GXTouch Analyzer and Qubit 3.0. RNA libraries were constructed with the KC Digital Ligation-specific Total RNA Library Kit. During library construction, random unique molecular identifier (UMI) tags were ligated to each RNA molecule to eliminate technical bias derived from PCR amplification. After enrichment of 200–500 bp fragments and Qubit quantification, sequencing was performed on the DNBSEQ-T7 platform in PE150 mode. In this study, fastp (version 0.23.2) was used for sequencing data quality control. Adaptor sequences were trimmed from raw reads. Reads with insert fragments shorter than 15 bp, poor quality or more than five N bases were subsequently filtered out. After evaluating base composition distribution and raw data quality, the integrity of initial RNA was assessed by analyzing the uniform distribution of reads across genes. Post-alignment UMI-based error correction and duplicate removal were implemented via gencore software to acquire accurate molecular sequences and gene expression profiles, with detailed procedures described below. First, reads were clustered based on mapping coordinates and UMI sequences; reads were grouped by both mapping positions and UMIs when available, while clustering was conducted solely according to mapping positions for UMI-free reads. Second, valid clusters with supporting read counts exceeding the predefined threshold were retained to generate consensus reads. Third, overlapping regions of paired-end reads were identified, and base scores within overlapping segments were calculated in light of base concordance and base quality. Fourth, weighted total scores of distinct nucleotides (A/T/C/G/N) at each base position were computed using quality values of all supporting reads. Finally, the consensus base was defined as the dominant nucleotide with a score higher than the preset threshold; if no dominant nucleotide existed, the corresponding base from the reference genome was adopted (where available).

### Analysis of differential expression of lncRNA

**Figure 1:**
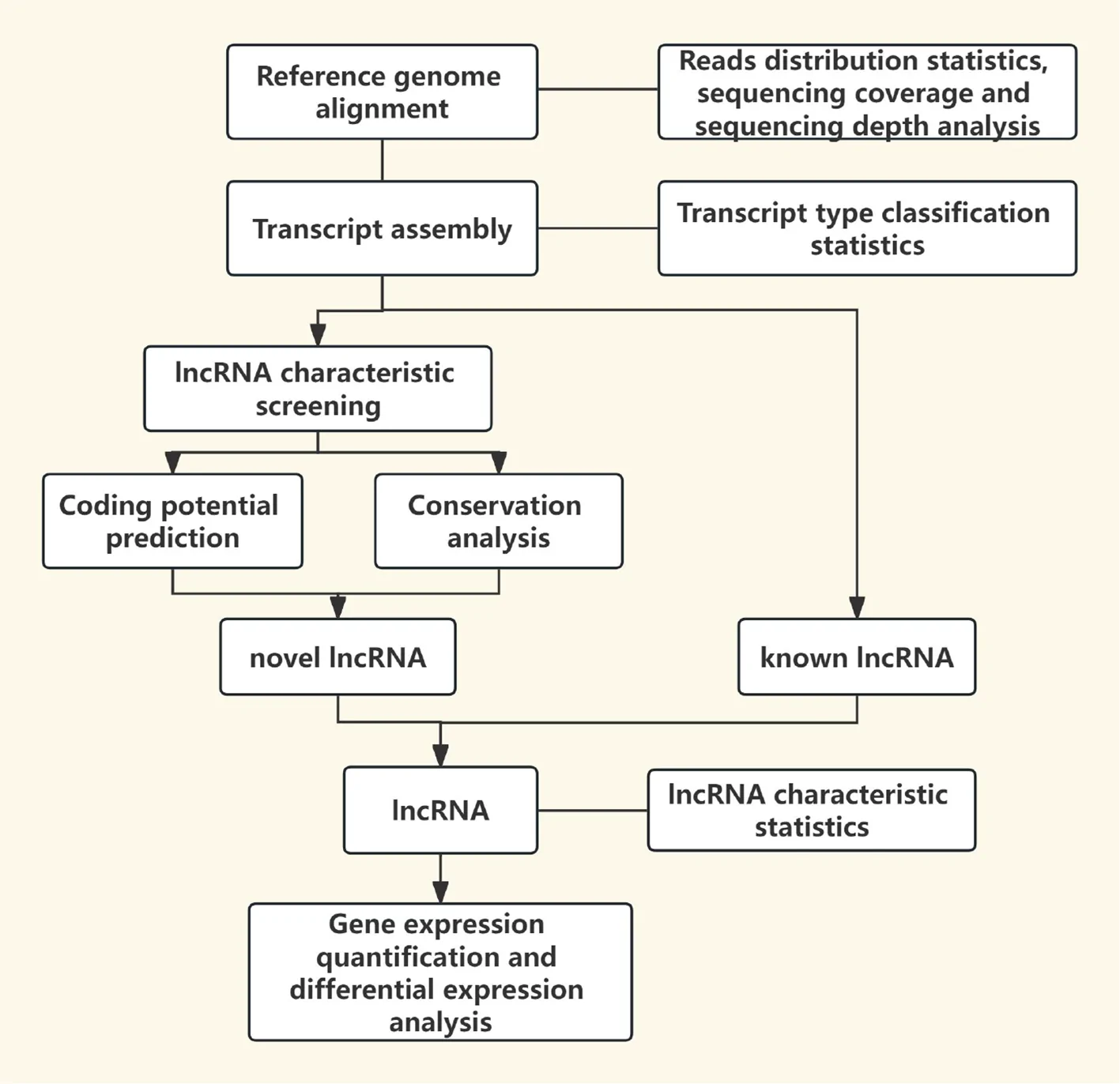
Analysis workflow for lncRNAs.

Based on the structural and expression characteristics of lncRNAs, strict screening criteria were set to obtain the transcript assembly results of StringTie. Based on the assembly results from Stringtie, two major steps of screening are carried out simultaneously. The first step is the basic screening (initial screening), and the second step is the screening based on its coding ability. The basic screening mainly consists of three parts: Step 1: Select transcripts with a length of at least 200 base pairs and an exon count of at least 2; Step 2: Calculate the read coverage for each transcript using stringtie, and select the transcripts with the minimum read coverage of at least 3. Step 3: Filter out non-lncRNA genes by comparing them with known lncRNA genes, and conduct position screening using the results of cuffcompare. Through the preliminary screening of lncRNAs and the screening according to coding ability, the number of novel lncRNAs was obtained. In addition, the known lncRNAs from the genomic annotation file were found, and the lncRNAs in this sample were identified through the comparison results. On the basis of the difference ratio |logFC| > 1 and the significance level pvalue < 0.05, combined with the gene expression comprehensive database Gene Expression Omnibus (GEO) and the Cancer Genome Atlas database (TCGA), and in conjunction with reading relevant literature, lncRNAs with significant expression differences were selected for subsequent research. The specific analysis workflow for lncRNAs (**Figure 1**) is as follows.

### Verification of the candidate lncRNA

RNA extraction solution, external reference solution and chloroform substitute were added and fully blended. The mixture was placed still and centrifuged at 12000 rpm for 10 min at 4 °C. The supernatant was collected and mixed with isopropanol, followed by incubation at −20 °C and re-centrifugation to harvest RNA precipitates. The precipitates were washed with 75% ethanol, air-dried and dissolved in RNA elution buffer. RNA concentration and purity were measured using a Nanodrop 2000 spectrophotometer. Subsequently, total RNA was reversely transcribed into cDNA in a 20 μL reaction system with custom gene-specific primers via a conventional PCR instrument. Quantitative real-time PCR was performed using SYBR Green reagent with three technical replicates for each sample. The reaction was initiated with pre-denaturation at 95 °C for 30 s. Amplification was carried out for 40 cycles at 95 °C for 15 s and 60 °C for 30 s. 2−ΔΔCT relative quantitative method was used to analysis results. During the sample verification process, GAPDH was used as the internal reference for correction. During the cell line verification process, CEL-MIR-39-3P was used as the external reference for correction. We selected CEL-MIR-39-3P from the nematode source as the exogenous reference. Traditional internal references (U6, GAPDH) are almost impossible to be encapsulated in exosomes, and there are differences in RNA recovery rates, resulting in quantitative deviations. Therefore, it can effectively correct the technical differences between samples.

### Diagnostic performance and prognostic analysis of candidate lncRNA

The relative expression levels of candidate lncRNAs were calculated, and ROC curves with corresponding AUC values were constructed based on our in-house cohort (36 paired RCC tumor and adjacent non-tumor tissues) to evaluate diagnostic performance. Kaplan–Meier survival analysis was further applied to assess the prognostic significance of candidate exosomal lncRNAs in RCC patients, and this survival analysis was conducted using public TCGA dataset rather than our small internal clinical cohort. Survival-related bioinformatics analyses were performed using the TCGA clear cell renal cell carcinoma (ccRCC) public cohort dataset, which was completely independent of our small plasma discovery cohort. All ccRCC patients in the TCGA dataset were split into a high-expression group (n=429) and a low-expression group (n=430) according to the median expression level of SLC16A1-AS1 as the cut-off value.

### Statistical analysis

Statistical analyses and graphs were generated with GraphPad Prism 11.0. Data are presented as mean ± standard deviation, with ≥3 biological and technical replicates. Normality and variance homogeneity were tested prior to comparison. Parametric tests (t-test, one-way/two-way ANOVA) were applied for normally distributed data with equal variance; non-parametric methods were used otherwise. All data in this study met parametric assumptions. Two-tailed P < 0.05 was set as the significance threshold, and P < 0.01 as highly significant.

## Results

### Characterization of exosomes in plasma

**Figure 2:**
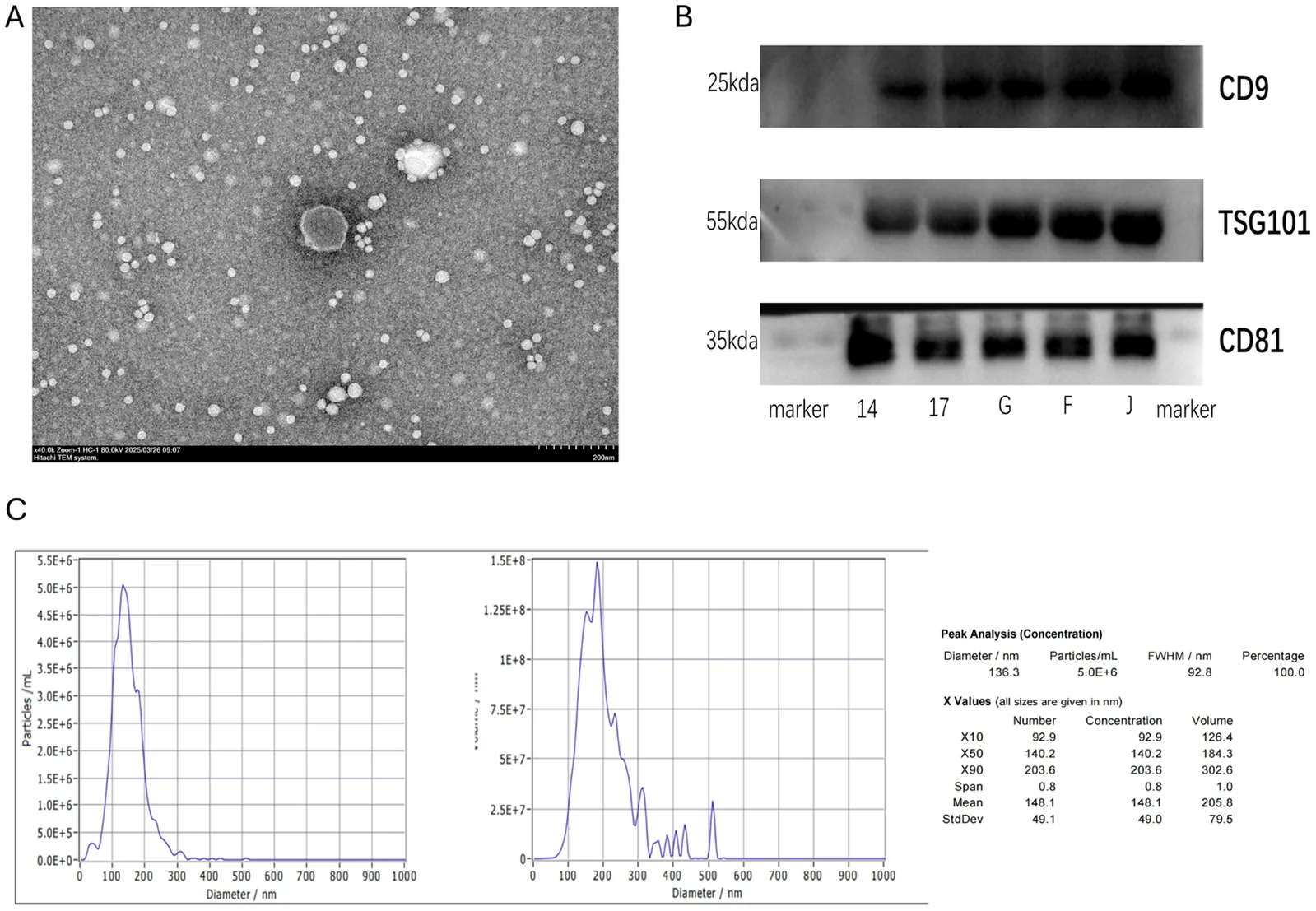
Characteristics of exosomes derived from plasma sources. **(A)** TEM detection of plasma exosomes. The image was acquired at ×40,000 magnification on a Hitachi HC-1 TEM operating at 80 kV, scale bar = 200 nm. **(B)** Representative WB banding patterns of characteristic markers of exosomes. **(C)** The quantity concentration and light intensity response distribution of exosomes were determined through NTA.

TEM revealed spherical or elliptical vesicles ranging from 30–140 nm, consistent with typical exosome morphology (**Figure 2A**).WB confirmed the expression of exosomal marker proteins CD9, TSG101 and CD81 (**Figure 2B**). NTA showed a unimodal particle size distribution of 30–200 nm, with a main peak at approximately 100 nm. The light intensity peak matched the concentration peak, confirming purity and intact morphology of isolated exosomes. The modal particle diameter was 136.3 nm, the mean particle size was 148.1 nm with a standard deviation of 49.1 nm. The particle concentration of exosomes was 5.0×10^6^ particles/mL (**Figure 2C**).

### Gene expression profiling and functional enrichment analysis

During the random sequencing process, the probability of long sequence genes being captured is higher than that of short sequence genes, which may lead to an overestimation of their expression levels. At the same time, the differences in sequencing depth among different samples will further interfere with the judgment of the true expression level of genes. The FPKM method can correct the deviations caused by gene length and sequencing depth, and the obtained gene expression levels are applicable for the comparison of expression differences among different samples. If a gene contains multiple transcripts, its sequencing coverage and expression level will be uniformly calculated based on all exons of the gene. Thus, the Fragments Per Kilobase Per Million Reads (FPKM) value was adopted as the indicator to quantify gene expression levels. The FPKM calculation method is as follows:


$$FPKM=\frac{total\;exon\;fragments}{mapped\;fragments(millions)\times exon\;length(KB)}$$

**Figure 3:**
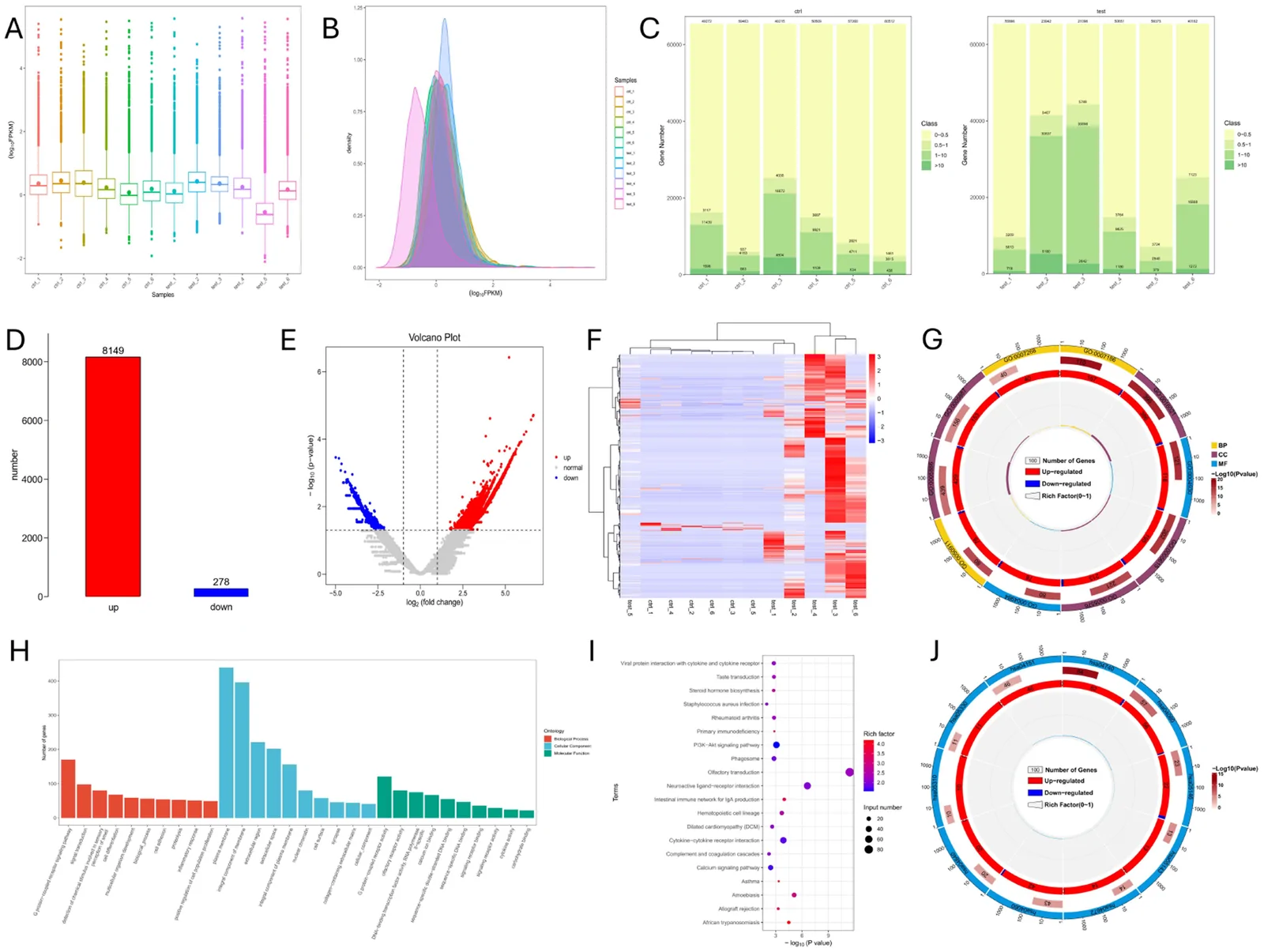
**(A)** Box plot: the x-axis represents sample names, and the y-axis denotes log_10_-transformed expression values. Each box displays five statistics (from top to bottom: maximum, upper quartile, median, lower quartile, minimum); **(B)** Density plot illustrating log_10_-transformed expression distribution of all transcripts; distinct colors denote different samples. The y-axis represents transcript proportion, the area under each curve equals 1, and curve peaks indicate concentrated expression levels. **(C)** Stacked bar chart classifies transcripts into four expression levels (color depth corresponds to rising expression). X-axis shows samples, y-axis indicates transcript counts of each expression group; **(D)** Bar chart: red for upregulated genes and blue for downregulated genes. A total of 8149 upregulated and 278 downregulated genes were identified via intergroup comparison; **(E)** Gray dots: non-differentially expressed genes; blue dots: downregulated DEGs; red dots: upregulated DEGs; **(F)** Hierarchical clustering heatmap of differentially expressed genes: Genes were clustered according to their expression levels. Red and blue denote high and low expression, respectively. X-axis: samples; Y-axis: differentially expressed genes. Dendrograms indicate sample and gene clustering. Obvious expression differences existed between groups. **(G)** Outer ring: GO terms and categories (yellow: BP, purple: CC, sky blue: MF), numbers denote enriched gene counts, color depth represents -log_10_(P Value). Middle ring: gene expression (red: upregulated, blue: downregulated). Inner ring: enrichment score (ratio of foreground to background genes); **(H)** GO enrichment bar plot of DEGs (BP/CC/MF), colors correspond to functional categories at top-right. DEGs are mainly enriched in plasma membrane, GPCR signaling transduction and GPCR activity; **(I)** KEGG enrichment shows DEGs enriched in PI3K-Akt, cytokine-receptor and neuroactive ligand-receptor interaction pathways, which may regulate RCC progression; **(J)** Outer ring: pathway names, numbers indicate enriched gene counts, color intensity represents -log10(P Value). Middle ring: red upregulated, blue downregulated. Inner ring: enrichment score (foreground/background gene ratio).

The expression levels of the transcripts standardized by the above methods were presented in box plots (**Figure 3A**), density distribution graphs (**Figure 3B**), and stacked bar charts to show their distribution patterns (**Figure 3C**). By comparing different groups, a total of 8,149 up-regulated genes and 279 down-regulated genes were obtained (**Figure 3D**). Volcano plots were utilized to intuitively illustrate the upregulated and downregulated differentially expressed genes (**Figure 3E**). Cluster analysis was performed on genes with similar or related expression patterns, which can be used to uncover the unknown functions of genes or to annotate the new functions of known genes; genes within the same cluster tend to have similar functions or jointly participate in the same metabolic pathways and signal regulation (**Figure 3F**). For Gene Ontology (GO) enrichment, prominent enriched terms fell into three categories: cellular component (CC), biological process (BP), and molecular function (MF). CC terms were dominated by membrane compartments including plasma membrane and integral membrane components; top BP entries encompassed G protein-coupled receptor (GPCR) signaling and general signal transduction; the primary MF term was GPCR activity. Collectively, these annotations indicate DEGs primarily mediate membrane-localized signal sensing and transmission in RCC cells (**Figure 3H**). The GO circle plot (**Figure 3G**) graphically summarizes functional classifications, gene counts and enrichment magnitudes of all DEG-enriched GO terms. KEGG pathway enrichment further identified tumor-relevant signaling cascades linked to RCC pathogenesis. The bubble plot (**Figure 3I**) displays high-ranking enriched pathways, where dot size corresponds to the number of enriched DEGs per pathway. Key enriched pathways included PI3K/Akt signaling, cytokine–receptor interaction, and neuroactive ligand–receptor interaction, implying DEGs drive RCC initiation and progression by modulating these oncogenic signaling axes. The KEGG circle diagram (**Figure 3J**) complements this visualization by systematically illustrating pathway identities, associated gene counts, and corresponding enrichment levels of DEGs.

### Analysis of high-throughput sequencing results of lncRNA

Based on the sequence characteristics of lncRNAs, we established strict screening conditions and used the StringTie software to complete transcriptome assembly. The screening was divided into two major steps: the first step was the basic screening, which consists of three steps (**Table 3**):Step 1: Select transcripts with a length of >= 200 bp and an exon number of >= 2; Step 2: Calculate the read coverage of each transcript using stringtie, and select the transcripts with the minimum read coverage of >= 3; Step 3: Filter out non-lncRNA genes by comparing with known non-lncRNA genes, and perform position screening using the results of cuffcompare.

**Table 3:** Initial screening results of novel lncRNA transcripts.

Select_Type	Assembled_Transcripts	Step1	Step2	Step3
Select_Num	395770	365396	79372	3455

**Figure 4:**
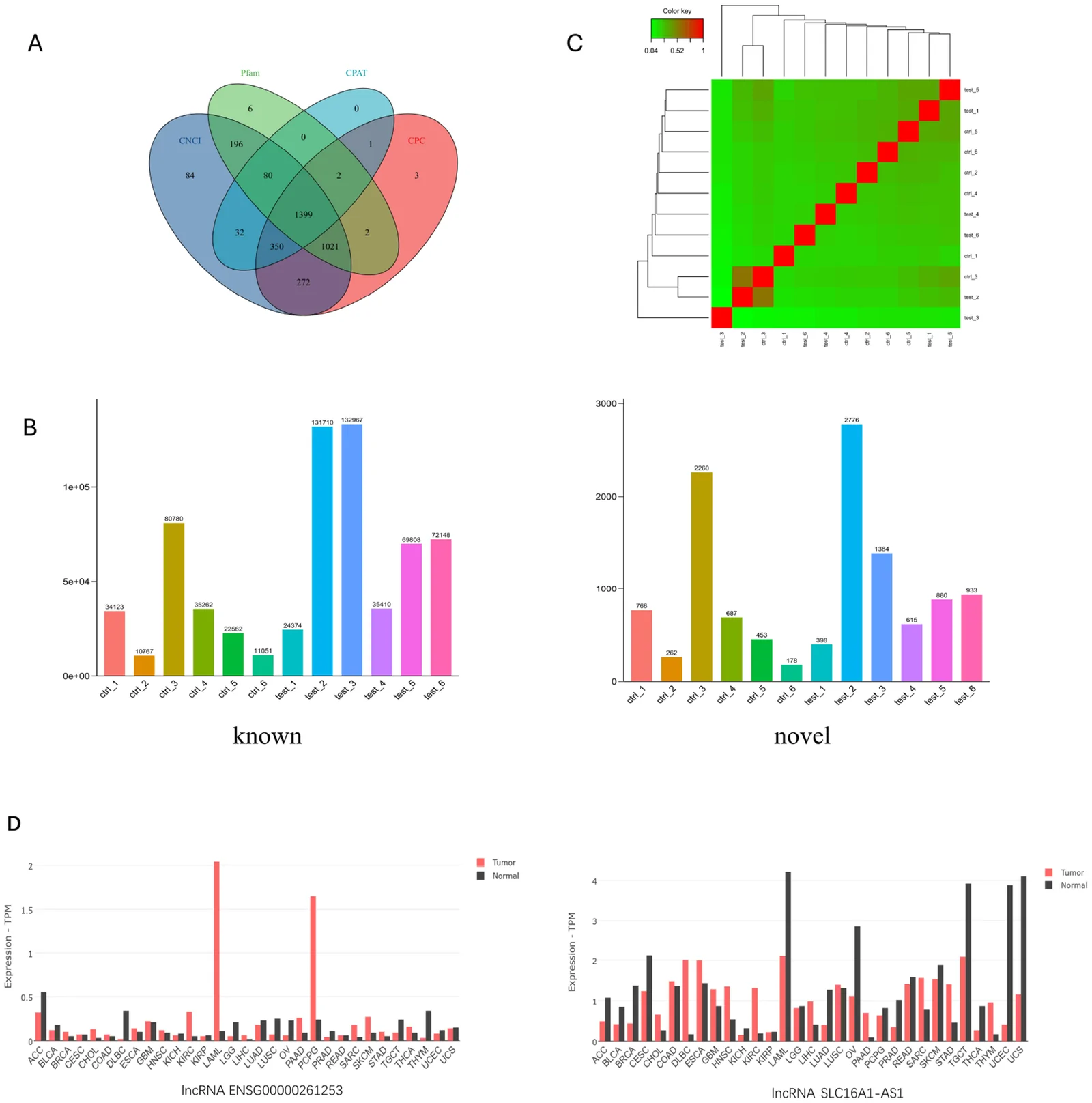
**(A)** Four prediction methods show consistency and specificity, with 1399 overlapping transcripts. These candidates have consistent features in CNCI, CPAT, CPC and Pfam, forming a high-confidence dataset; **(B)** Two-panel plot showing counts of known and novel lncRNAs in control (Ctl_1-Ctl_6) and test (test_1-test_6) groups. Left panel: known lncRNAs (Y-axis: 0-1×10^5^); right panel: novel lncRNAs (Y-axis: 0-3000); **(C)** Hierarchical clustering plot of transcript expression. Color bar (green-red) indicates low-to-high sample-to-sample correlation. Branches denote sample clustering; shorter branches mean higher similarity, longer branches lower similarity. **(D)** The expression distribution (quantified as TPM, Transcripts Per Million) of two candidate lncRNAs (ENSG00000261253, SLC16A1-AS1) in tumor and paired adjacent normal tissues from TCGA and GEO public transcriptome databases.

The second step was the screening of the protein-coding ability of the transcripts, which was carried out from four dimensions: CPC, CNCI, Pfam protein domains, and CPAT. The intersection of the lncRNAs predicted by the four tools was taken as the novel predicted lncRNAs. The results obtained by these four methods are consistent and specific. The transcripts predicted by all of them total 1399 (as shown in the top overlapping area of the figure), and the coding potential features CNCI, CPAT, CPC, and Pfam of these transcripts are all consistent, making them the high-confidence candidate set of this study. (**Figure 4A**).

After obtaining the number of novel lncRNAs, we identified the known lncRNAs from the genomic annotation file and extracted the lncRNAs present in the sample based on the comparison results. The statistics are shown in **Figure 4B**, and the correlation heatmap of lncRNA differential expression is presented in **Figure 4C**. We selected the lncRNAs with significant expression differences based on the two criteria: the difference ratio |logFC| > 1 and the significance level p value < 0.05. The results of the significant differences are as follows (**Table 4**):

**Table 4:** Statistical results of differential expression of lncRNA.

Compare group	Total differentially expressed	Up	Down
ctrl 1–6 vs test 1-6	10325	9853	472

To improve the reliability of subsequent experimental validation, we integrated fold change, statistical significance and screened candidate lncRNAs by combining datasets from the GSE and TCGA databases. Firstly, based on our NGS data, we selected the five most significantly differentially expressed candidate lncRNAs. Subsequently, we submitted these candidate lncRNAs to the TCGA and GEO (GSE) databases for secondary screening (**Figure 4D**). Finally, lncRNA ENSG00000261253, lncRNA FRRS1L, lncRNA LINC02035, lncRNA DNAJC7, and lncRNA SLC16A1-AS1 were selected for the next verification experiment. After reviewing the data, it was found that FRRS1L and DNAJC7 are protein-coding genes, which is inconsistent with our sequencing results. Therefore, the data of lncRNA FRRS1L and lncRNA DNAJC7 should be regarded as merely for reference.

### Verification of the candidate lncRNA

**Figure 5:**
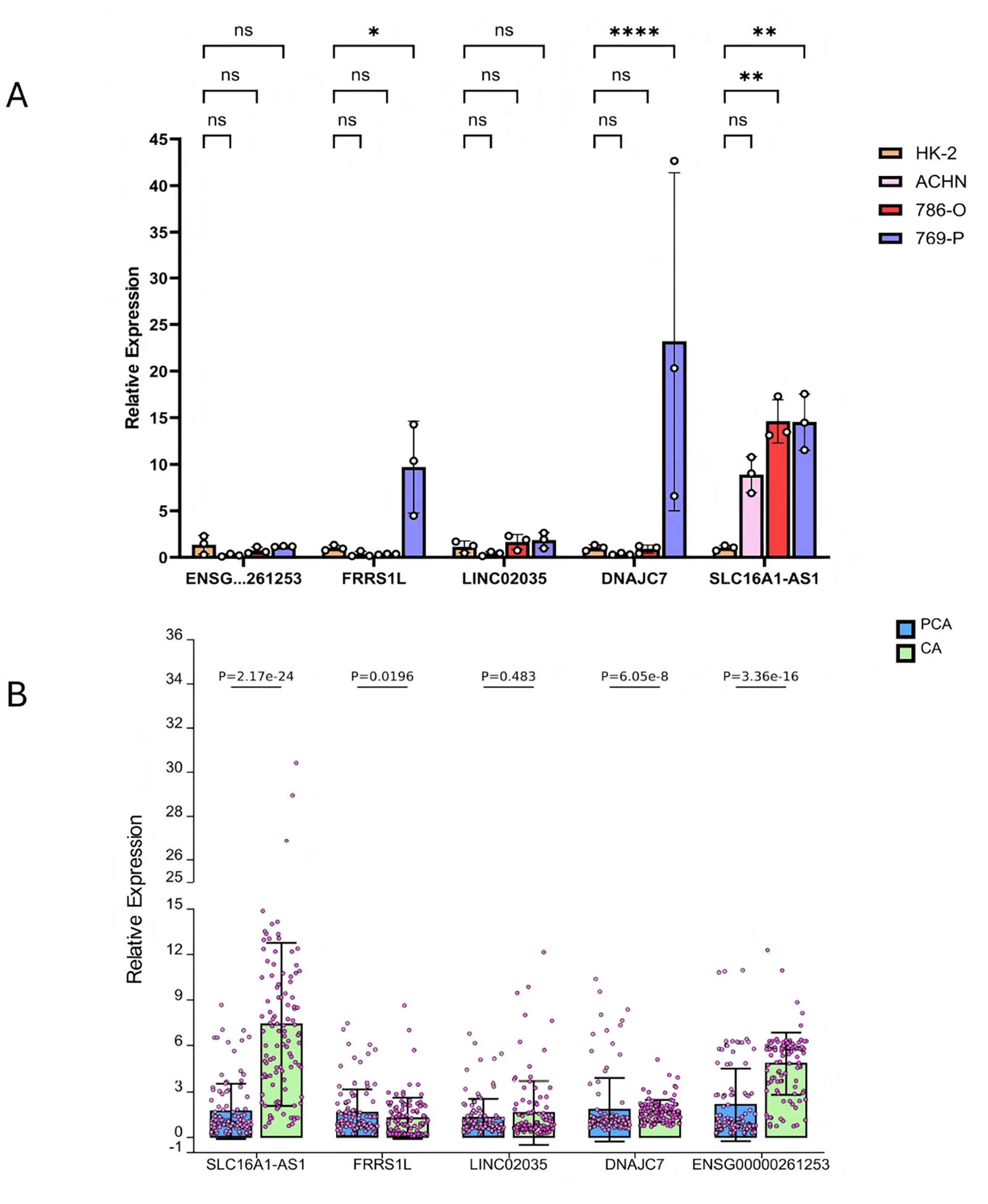
**(A)** X-axis: target molecule; Y-axis: relative expression. Colored bars indicate different cell lines. Error bars represent mean ± SEM, dots show raw data. ns, no significance vs HK-2 (p ≥ 0.05); *p<0.05, **p<0.01, ****p<0.0001. Plasma-exosomal lncRNA SLC16A1-AS1 is highly expressed in RCC cell lines, especially in 786-O and 769-P; **(B)** X-axis: target molecule; Y-axis: relative expression. Colored bars represent different tissues. Error bars denote mean ± SEM, dots show raw data. SLC16A1-AS1 exhibits the most significant upregulation in RCC tissues. PCA, Paired adjacent normal renal tissues; CA, renal cell carcinoma tissues.

The relative expression of five target plasma exosomal lncRNAs in three renal cancer cell lines (ACHN, 786-O, 769-P) and one normal renal tubular epithelial cell line (HK-2) is shown in **Figure 5A**. Among them, the plasma exosomal lncRNA SLC16A1-AS1 was highly expressed in the renal cancer cell lines to the greatest extent, especially in the 786-O and 769-P renal cancer cell lines, with significant high expression (**, P < 0.01), and the difference was statistically significant. In 36 pairs of renal cell cancer patient tumor and adjacent tissues, the expression levels of five plasma exosomal lncRNAs were verified (**Figure 5B**). It was found that the expression level of plasma exosomal lncRNA SLC16A1-AS1 showed the most significant difference.

### Diagnostic performance and prognostic analysis of candidate lncRNAs

**Figure 6:**
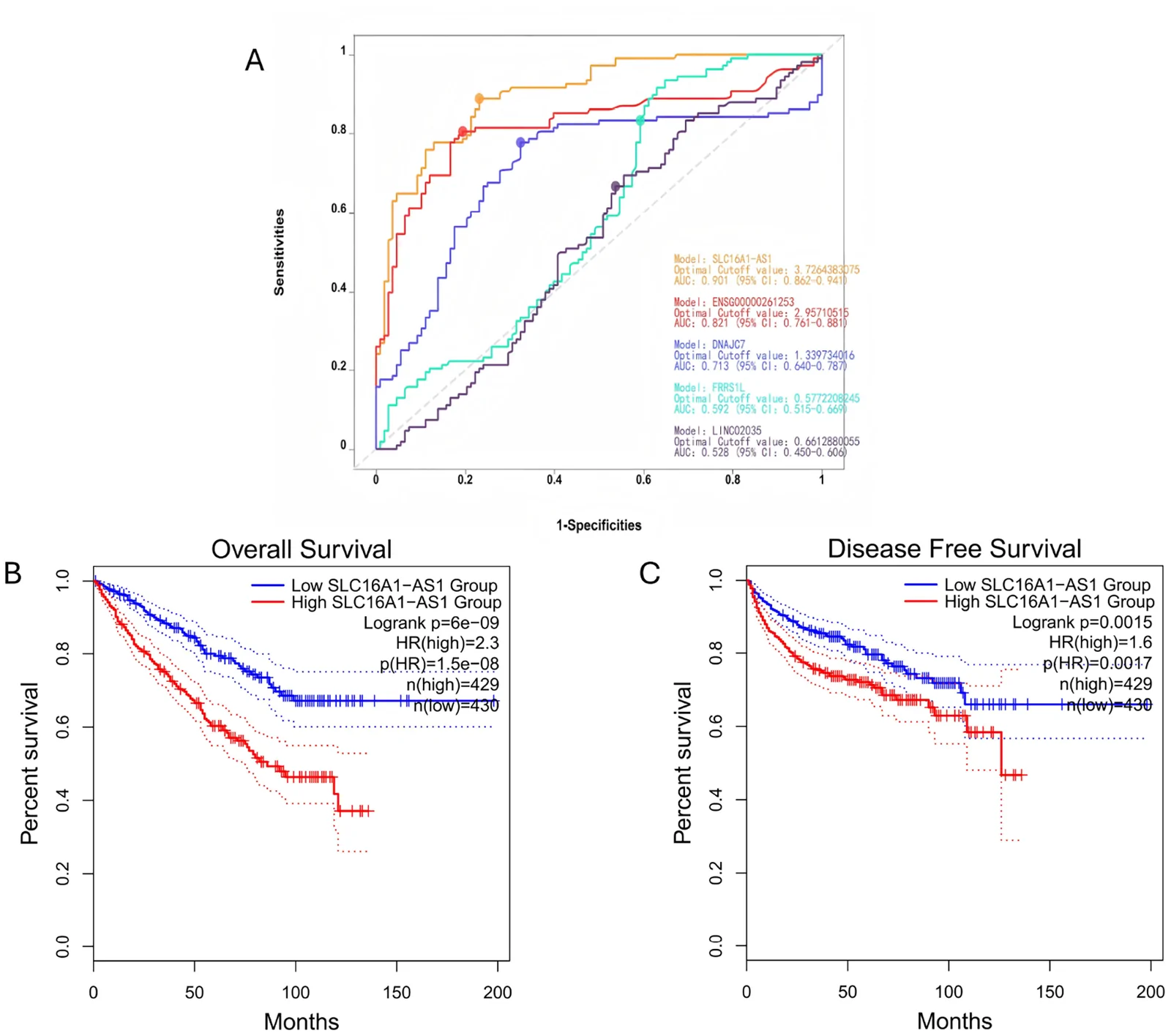
**(A)** ROC curves for three candidate lncRNAs and two protein-coding transcripts in distinguishing RCC from healthy controls. The AUC values, optimal cut-off values and 95% confidence intervals for each biomarker are displayed within the plot.; **(B)** Kaplan-Meier OS curves of RCC patients derived from the TCGA cohort, stratified by SLC16A1-AS1 expression level. Patients were divided into high-expression group (n = 429) and low-expression group (n = 430). Log-rank test p = 6e-09, HR = 2.3. Dotted lines represent 95% confidence intervals.; **(C)** Kaplan-Meier DFS curve of RCC patients from the TCGA cohort, stratified by SLC16A1-AS1 expression level. The high-expression group contained 429 patients and the low-expression group contained 430 patients. Log-rank test p = 0.0015, HR = 1.6. Dotted lines represent 95% confidence intervals.

The performance of the target lncRNA for diagnosing renal cell cancer was evaluated using the ROC curve (**Figure 6A**). The results showed that the plasma exosomal lncRNA SLC16A1-AS1 performed the best, with an AUC value of 0.901 (95% confidence interval: 0.862 - 0.943), far exceeding the random level, indicating that it has strong application value for diagnosing renal cell cancer. Kaplan–Meier analysis OS (**Figure 6B**) and DFS (**Figure 6C**) were conducted for lncRNA SLC16A1-AS1. By using public TCGA dataset, the samples were divided into a high-expression group (n=429) and a low-expression group (n=430). The results may showed that patients with high expression of lncRNA SLC16A1-AS1 had a worse prognosis. These findings may indicate that the high expression of lncRNA SLC16A1-AS1 is significantly associated with poor overall survival and can serve as a potential adverse prognostic marker.

## Discussion

In this study, we utilized NGS to identify differentially expressed exosomal lncRNAs between patients with RCC and healthy individuals. After reviewing the related data, it was found that FRRS1L and DNAJC7 are protein-coding genes, which is inconsistent with our sequencing results. Therefore, the data of lncRNA FRRS1L and lncRNA DNAJC7 should only be regarded for reference. Eventually, lncRNA SLC16A1-AS1 was identified as potentially having a biomarker function. Subsequent cell line and tissue validation analyses further confirmed that SLC16A1-AS1 in plasma exosomes was significantly upregulated in RCC patients. The diagnostic performance was quantified through ROC curve and AUC analysis. The results indicated that plasma exosomal lncRNA SLC16A1-AS1 showed a good AUC value, suggesting its good discriminatory ability in differentiating patients with RCC from healthy control groups. Additionally, Kaplan-Meier survival analysis was used to explore the prognostic significance of this molecule in RCC patients. The results showed that increased expression of plasma exosomal lncRNA SLC16A1-AS1 was associated with poor survival outcomes in RCC patients, preliminarily indicating its potential prognostic predictive value. Prior mechanistic work by Li et al. demonstrated oncogenic activity of SLC16A1-AS1 in RCC cell models: SLC16A1-AS1 was upregulated in RCC cell lines, and its knockdown suppressed cell proliferation and migration while triggering ferroptosis via the miR-143-3p/SLC7A11 axis, supporting its functional involvement in RCC progression (19). However, that study was restricted to *in vitro* cellular assays; it did not assess circulating exosomal SLC16A1-AS1, diagnostic performance by ROC analysis, or patient-level survival associations. Our study was the first to focus on the lncRNA SLC16A1-AS1 in the plasma exosomes of RCC patients, systematically verified its abnormal expression characteristics, and preliminarily evaluated its diagnostic efficacy and prognostic potential, thus filling the current gap in the research on this molecule as a circulating exosome biomarker and clinical prognosis indicator in RCC.

### The advantages of plasma exosomal lncRNA and application prospect

Compared with traditional tissue biopsy, plasma exosomal lncRNAs possess distinct superiorities. Firstly, they enable non-invasive detection via peripheral blood sampling, avoiding the trauma and risks of tissue biopsy, which facilitates early diagnosis, regular follow-up and prognostic monitoring (20). Secondly, plasma exosomal lncRNAs can dynamically reflect tumor biological status, allowing real-time monitoring of disease progression and therapeutic response to optimize clinical treatment strategies (21, 22). Thirdly, the membranous structure of exosomes protects internal lncRNAs from degradation, ensuring high stability and detection sensitivity, and conferring favorable reliability and repeatability for clinical application (23). Plasma exosomal lncRNAs exhibit great application potential in RCC management. They can serve as promising biomarkers for early RCC screening to elevate early diagnostic efficiency before clinical symptoms emerge (24). The expression profiles of these molecules also contribute to the formulation of individualized therapeutic regimens and improve clinical outcomes (25). Accumulated studies have verified that exosomes are reliable tools for early diagnosis, prognostic judgment and therapeutic surveillance of RCC, which can deliver various therapeutic agents to achieve precise personalized treatment (26). Moreover, exosomal lncRNAs serve as reliable indicators for assessing patient survival and tumor recurrence risk. They also help refine current prognostic evaluation systems and optimize immunotherapeutic strategies. In addition, aberrantly expressed lncRNAs are considered promising therapeutic targets for RCC. Multiple studies have confirmed that specific lncRNAs mediate malignant biological behaviors of RCC by modulating relevant signaling pathways, thereby offering novel strategies for targeted therapy. For instance, exosome-transmitted LncARSR facilitates RCC progression via activating the STAT3 pathway to induce macrophage polarization, and the LncARSR-miR34/miR449-STAT3 axis serves as a promising therapeutic target against RCC (27).

In conclusion, with continuous advancement of relevant research and detection technologies, plasma exosomal lncRNAs will exert increasingly vital roles in early diagnosis, precise treatment and prognostic evaluation of RCC.

### The role of exosomal lncRNAs from different sources in the development of RCC

Plasma exosomal lncRNAs participate in RCC pathogenesis via multiple biological mechanisms. Acting as core intercellular communicators in the tumor microenvironment (EMT), exosomes remodel the microenvironment of distant organs and promote tumor cell colonization (28). Exosomal lncRNA GIHCG is significantly upregulated in RCC and correlated with tumor size, lymph node metastasis and TNM stage, serving as a candidate diagnostic biomarker. In addition, the expression level of exosomal lncRNA GIHCG is elevated in lung cancer patients and can be used to distinguish lung cancer (29). Tumor-derived signals activate endothelial cells to facilitate angiogenesis (30). In RCC, plasma exosomes contain a large amount of azurocidin protein (AP), which plays a role in increasing vascular permeability. Therefore, it was observed that tumor-derived plasma exosomes can induce changes in the phenotype of endothelial cells, thereby leading to the destruction of vascular morphology (31, 32). lncRNA PANTR1 plays a relevant role in RCC by influencing cell apoptosis and angiogenesis (33). Exosomes facilitate tumor immune escape via inhibiting natural killer (NK) cell cytotoxicity and immune cytokine secretion, whereas RCC-derived exosomal lncRNAs suppress NK cell activity to enable tumor cells to escape immune surveillance (34, 35). Tumor microenvironment contributes substantially to RCC invasion and metastasis through impaired cell adhesion and gene expression reprogramming (36). Plasma exosomes from RCC patients deliver functional lncRNAs to induce EMT and accelerate metastatic progression (37).

### Limitations of the study and future prospects

Nevertheless, it is worth noting that this study is still in the exploratory stage, and several limitations still exist in the present study. First, no consensus has been established on the isolation, extraction and analytical methods of circulating exosomal lncRNAs in RCC, leading to inconsistent results across different researches. Second, these molecules lack sufficient tumor specificity. Certain lncRNAs are abnormally expressed in various malignancies, which restricts their application as exclusive diagnostic markers for RCC. Third, high detection costs such as high-throughput sequencing hinder large-scale clinical promotion. In addition, the sample size of this study is relatively small, the limited discovery cohort and lack of an independent plasma validation cohort, which need further large-sample and multicenter researches are required to validate our findings. This study only conducted differential expression analysis without relevant functional verification. Further *in vitro* and *in vivo* experiments are needed to clarify the biological functions of candidate lncRNAs in regulating proliferation, apoptosis, migration and invasion of renal cell carcinoma cells. More sensitive and accurate detection techniques also need to be developed. Given the complexity of tumor heterogeneity, combined detection of lncRNAs with proteins or microRNAs can effectively improve the screening efficiency of RCC.

Joint efforts are required to unify research protocols and develop high-sensitivity and high-specificity detection technologies. In general, plasma exosomal lncRNAs possess promising prospects for early diagnosis of RCC. The limitations and prospective suggestions proposed in this study are expected to facilitate further in-depth investigations and accelerate the clinical transformation of this novel biomarker.

## Data Availability

The datasets presented in this study can be found in online repositories. The names of the repository/repositories and accession number(s) can be found in the article/**Supplementary Material**.
